# Ilexsaponin A attenuates ischemia-reperfusion-induced myocardial injury through anti-apoptotic pathway

**DOI:** 10.1371/journal.pone.0170984

**Published:** 2017-02-09

**Authors:** Shuang-Wei Zhang, Yu Liu, Fang Wang, Jiao Qiang, Pan Liu, Jun Zhang, Jin-Wen Xu

**Affiliations:** 1 The second affiliated hospital of Guangzhou Medical University, Changgang east road, Guangzhou, China; 2 School of Chinese Pharmaceutical Science, Guangzhou University of Chinese Medicine, University Town, Guangzhou, China; 3 Basic Medical College, Guangzhou University of Chinese Medicine, University Town, Guangzhou, China; Virginia Commonwealth University, UNITED STATES

## Abstract

The protective effects of ilexsaponin A on ischemia-reperfusion-induced myocardial injury were investigated. Myocardial ischemia/reperfusion model was established in male Sprague–Dawley rats. Myocardial injury was evaluated by TTC staining and myocardial marker enzyme leakage. The in vitro protective potential of Ilexsaponin A was assessed on hypoxia/reoxygenation cellular model in neonatal rat cardiomyocytes. Cellular viability and apoptosis were evaluated by MTT and TUNEL assay. Caspase-3, cleaved caspase-3, bax, bcl-2, p-Akt and Akt protein expression levels were detected by western-blot. Ilexsaponin A treatment was able to attenuate the myocardial injury in ischemia/reperfusion model by reducing myocardial infarct size and lower the serum levels of LDH, AST and CK-MB. The in vitro study also showed that ilexsaponin A treatment could increase cellular viability and inhibit apoptosis in hypoxia/reoxygenation cardiomyocytes. Proapoptotic proteins including caspase-3, cleaved caspase-3 and bax were significantly reduced and anti-apoptotic protein bcl-2 was significantly increased by ilexsaponin A treatment in hypoxia/reoxygenation cardiomyocytes. Moreover, Ilexsaponin A treatment was able to increase the expression levels of p-Akt in hypoxia/reoxygenation cellular model and myocardial ischemia/reperfusion animal model. Coupled results from both in vivo and in vitro experiments indicate that Ilexsaponin A attenuates ischemia-reperfusion-induced myocardial injury through anti-apoptotic pathway.

## Introduction

Myocardial ischemia reperfusion injury is one of the leading causes of death worldwide. Although early reperfusion therapy with thrombolytic drugs in myocardial infarction (MI) patients could reduce myocardial infarction size, this therapy tends to induce ischemia-reperfusion (IR) injury [1–3]. Various pharmacological agents have been developed to reduce IR injury, however, none of them have been used for MI patients in clinical practice [3–5]. Thus, further studies are needed to find novel drugs to combat myocardial IR injury.

*Ilex pubescens* Hook.et Arn. is an evergreen shrub belonging to Aquifoliaceae. Its roots named Mao Dong Qing (MDQ) in Chinese are commonly used to treat cardiovascular diseases in south China. Several saponins have been isolated from the roots of Ilex pubescens and some of them have been shown to have anti-inflammatory and anti-tumor effects [6–8]. However, little is known about the cardiovascular protective effects of these saponins from the roots of *Ilex pubescens*.

Apoptosis, a highly regulated and evolutionarily conserved process, is activated by the specific signaling cascades. Numerous studies have proved that apoptosis plays a key role in acute myocardial ischemia-reperfusion injury [9–10]. Apoptosis can be induced via activation of a death domain-containing receptor located at the plasma membrane. For instance, TNF-α is rapidly released from resident mast cells and macrophages in response to myocardial ischemia and then induces cell apoptosis by binding to TNF receptor [11]. Saponins derived from the root of *Ilex pubescens* have been shown lower TNF-α level in the carrageenan-injected paw tissues in rats [12]. We hypothesize that saponins from the root of *Ilex pubescens* might have protective effects on ischemia-reperfusion-induced myocardial injury rats. Thus, present study investigates the protective effect of Ilexsaponin A from MDQ on ischemia-reperfusion-induced myocardial injury in IR rats and in hypoxia/reoxygenation cardiomyocytes.

## Materials and methods

### Plant materials

The raw materials of MDQ were purchased from Caizhiling Chinese Herbal Slice Co., Ltd., Guangzhou, China. These materials were authenticated by Prof. Jingsong Zhou of Guangzhou University of Chinese Medicine. The authenticated voucher specimens were kept in the School of Chinese Medicine, Guangzhou University of Chinese Medicine.

### Preparation and chemical profiling analysis of Ilexsaponin A

**Fig 1:**
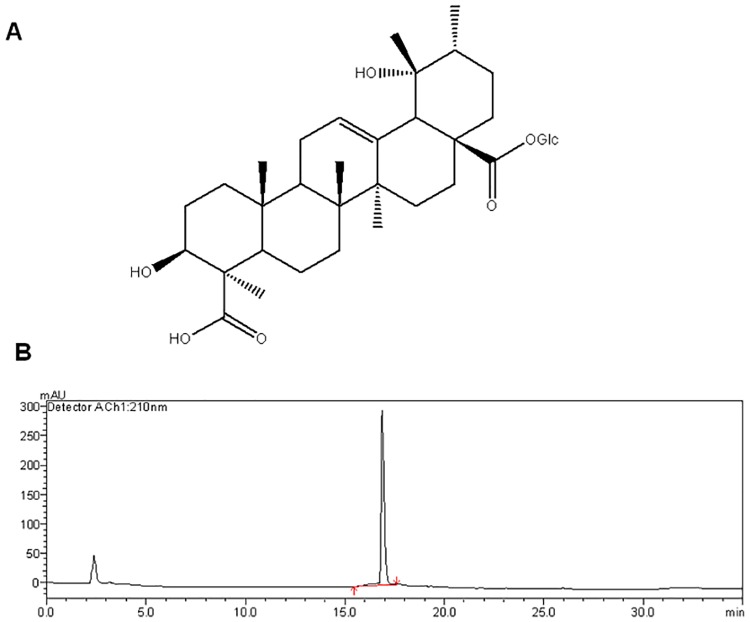
HPLC Chromatographic analysis and the chemical structure of Ilexsaponin A. A. Chemical structure of Ilexsaponin A B. HPLC Chromatographic analysis of Ilexsaponin A

Dried powders of the root(5000g) of *Ilex pubescens* Hook.et Arn *(I*. *pubescens*) were extracted with 70% aqueous ethanol (25000 mL) for 3 times at room temperature. The extracting solution was filtered and the combined filtrate was evaporated by rotary evaporator until without alcohol smell. Then the residue was successively partitioned with chloroform (5000 mL), ethyl acetate (5000 mL), *n*-butanol (5000 mL, saturated with water). The chloroform extract, ethyl acetate extract, and *n*-butanol extract were collected respectively. The *n*-butanol extract (258 g) was subjected to a silica gel colum (200–300 mesh; CHCl3-CH3OH, 100:0→0:100) to afford 7 fractions (A1-A7). Fraction A4 was separated on a Sephadex LH-20 colunm (CHCl_3_-CH_3_OH, 1:1), followed by HPLC (CH_3_OH-H_2_O, 65:35) to afford ilexoside A (23.2 mg). And it’s structure was established by a combination of spectroscopic methods, including HR-ESI-MS, IR, UV, ^1^H-NMR and ^13^C-NMR spectra (S1 and S2 Figs), and comparison the data with those in the literature [13]. Its purity detected by HPLC (Fig 1), was greater than 95% and reserved after freeze drying. Ilexoside A was dissolved by DMSO and diluted to corresponding concentrations by saline.

### Animals

Animal experiments were approved by the Animal Research Center of Guangzhou University of Chinese Medicine. Male Sprague–Dawley rats weighing 280–320 g were obtained from Laboratory Animal Center, Guangzhou University of Chinese Medicine. Animals were kept in pairs and under a 12-hour light/dark cycle started at 8:00 AM at a room temperature of 22–24°C and relative humidity of 50–55%, with free access to regular rodent chow and water. All experimental procedures were performed in accordance with the Guidelines of Animal Experiments from the Committee of Medical Ethics, National Health Department of China. Rats were randomly divided into four groups of eight animals: sham operation group (SHAM), ischemia/reperfusion injury group (I/R), Low dose of ilexsaponin A treatment group (AL,10mg/kg), High dose of ilexsaponin A treatment group (AH, 40mg/kg). Animals received humane care and were sacrificed under anesthesia with xylazine/ketamine (0.5 mL of xylazine [2 mg/mL] and 1 mL of ketamine [50 mg/mL]) at a dosage of 1.5 mL/100 g body weight.

### Myocardial ischemia/reperfusion procedure

The rats were anesthetized by 3% pentobarbital sodium (50mg/kg). Myocardial ischemia was made by exteriorizing the heart through a left thoracic incision and placing a 6.0 silk suture to make a silicone rubber venous cannula around the left anterior descending coronary artery. After half an hour, the venous cannula was removed to allow reperfusion of the ischemic myocardium for 4h. Ischemia was confirmed by myocardial blanching and electrocardiographic evidence of injury (widening of QRS complex and elevation of ST segment). Sham operation was made under the same surgical procedures except that the suture passed under the left coronary artery was left untied. Ilexsaponin A was given 10 min before ligating the left anterior descending coronary artery.

### Determination of myocardial infarction

At the end of 4h reperfusion, myocardial infarction was determined by means of a TTC staining and a digital imaging system [14]. Myocardial infarct size (IS) and area at risk (AAR) were determined as described previously [14]. Briefly, at the end of reperfusion period, the hearts were firstly perfused with Evan's blue at a constant pressure (80 mmHg). Then, the left ventricle was cut into 2–3 mm slices from the apex to the base. The perfused myocardium was stained blue, whereas the AAR remained unstained. The unstained myocardium was incubated for 30 min at 37°C in TTC (1% in 0.1 mol/L phosphate buffer, pH 7.4). The non-infarcted myocardium was deep red, in contrast to the pale white of the infarcted myocardium.

### Determination of serum CK-MB, LDH, AST

Blood received from the trunk was put into syringes, maintained at room temperature for half an hour and then centrifuged at 4,500g, 4°C, for 15 min. Serum levels of CK-MB, LDH and AST were assayed using commercial kits (Jiancheng Bioengineering Institute, Nanjing, China).

### Cell culture and hypoxia/reoxygenation model

Hearts were harvested from 1-3-day-old rat. Primary culture of neonatal rat cardiomyocytes were performed as described previously [15]. Isolated cardiomyocytes were seeded onto cell culture flask and cultured at 37°C in humid air with 5% CO_2_. The culture medium was replaced with fresh DMEM medium after 48 h and cells were further cultured for 24 h. Cells were incubated in a hypoxic/ischemic chamber also known as the Modular Incubator Chamber (MIC-101, Billups-Rothenberg, USA) at 37°C for 4 h in a humidified atmosphere of 5% CO_2_ and 95% nitrogen. For the reoxygenation process the cells were superfused in DMEM supplemented with 10% fetal calf serum at 37.0°C under 5% CO_2_ incubation for 4 h.

### Determination of cell viability and cellular apoptosis

The cellular viability was determined by MTT assay and apoptosis was analyzed by performing TUNEL assay using the in situ cell death detection kit according to the manufacturer’s instructions (Roche) [16]. The apoptotic index was expressed as the number of positively stained apoptotic cardiomyocytes/the total number of cardiomyocytes. In order to investigate the protective effect of Ilexsaponin A on hypoxia/reoxygenation model, cardiomyocytes were incubated with Ilexsaponin A at 10, 50, 250 μg/ml for 24h before hypoxia-reoxygenation treatment.

### Western blot analysis

Western blot analysis was performed as previously described [17]. Briefly, the cardiomyocytes samples were lysed in lysis buffer on ice for 20 min. The lysates then were centrifuged at 4°C for 15min at 12,000 rpm. After quantitation of protein concentration, 30 μg of total protein was separated by SDS-PAGE and then transferred to a polyvinylidene difluoride membrane (Millipore, USA). Antibodies used were as follows: caspase-3 (rabbit polyclonal, 9662, Cell Signaling Technology, USA), cleaved caspase-3 (rabbit polyclonal, 9661, Cell Signaling Technology, USA), bcl-2 (rabbit polyclonal, ab59348, Abcam), bax (rabbit polyclonal, 2772, Cell Signaling Technology, USA), p-Akt (rabbit polyclonal, 4060, Cell Signaling Technology, USA), Akt (rabbit polyclonal, 9272, Cell Signaling Technology, USA) and actin (rabbit polyclonal, 4970, Cell Signaling Technology, USA). Primary and secondary antibodies were incubated with the membranes by standard technique. Immunodetection was accomplished using enhanced chemiluminescence. Chemiluminescence was acquired with a quantitative digital imaging system (Quantity One; Bio-Rad, Hercules, CA) with provision to check for saturation. Overall emitted photons were quantified for each band, particularly for loading controls, which were homogeneously loaded.

### Statistical analysis

Data are presented as the mean ± standard deviation. Statistical analysis for the experimental groups was performed using SPSS for Windows version 13.0. Differences among groups were compared with one-way analysis of variance (ANOVA) followed by the Dunnett's test. Differences were considered statistically significant when P<0.05.

## Results

### The effects of Ilexsaponin A on myocardial infarct size in myocardial ischemia/reperfusion rats

**Fig 2:**
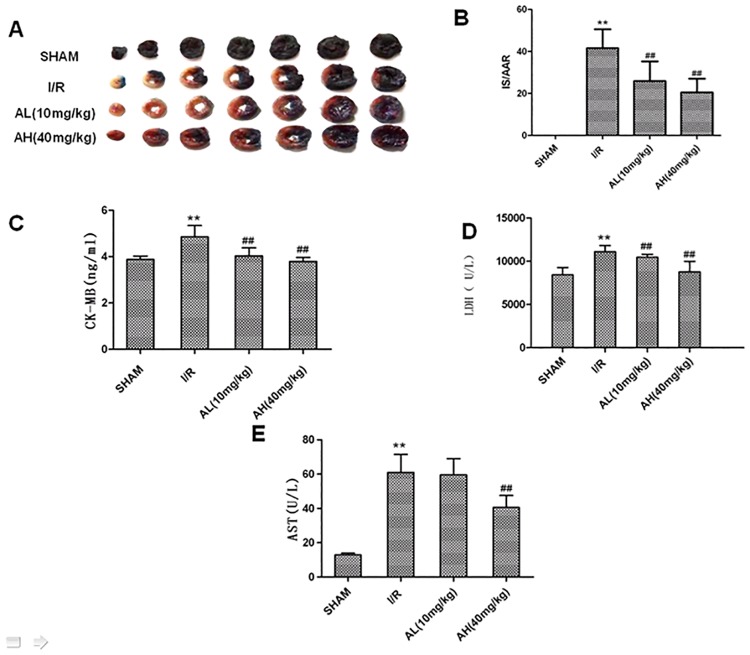
The effect of Ilexsaponin A treatment on infarct size and myocardial marker enzyme leakage in injured hearts induced by ischemia/reperfusion. A. Representative photographs of heart sections. Blue-stained region indicates non-ischemic, normal region; red-stained region indicates ischemic/reperfused but not infarcted region; and negative-stained region indicates ischemic/reperfused infarcted region. B. Quantification of myocardial infarct size expressed as percentage of area-at-risk(AAR). C. Serum CK-MB level D. Serum LDH level E. Serum AST level; AL: ilexasaponin A (10mg/kg), AH: ilexasaponin A (40mg/kg), P<0.05 versus SHAM (**) and versus I/R (##). Bars represent SD; n = 6–8 hearts per /group.

The IS/AAR ratio were 41.55%±8.99% in IR group rats. Both high and low dose of Ilexsaponin A treatment were able to reduce the myocardial infarct size, since the IS/AAR ratio were significantly lower in EL and EH group rats. The protective effects of Ilexsaponin A on myocardial ischemia/reperfusion rats seemed to be dose dependent, as the IS/AAR ratio in EL and EH group rats were 25.89%±9.33% and 20.49%±6.55% respectively (Fig 2A and 2B).

### The effects of Ilexsaponin A on serum dehydrogenase (LDH), aspartate transaminase (AST) and creatinine kinase-MB (CK-MB) in myocardial ischemia/reperfusion rats

In line with the results of myocardial infarct size, the levels of CK-MB, LDH and AST were significantly higher in I/R group than in SHAM group (Fig 2C, 2D and 2E). The levels of serum CK-MB were significantly reduced in EL and EH group. High dose of Ilexsaponin A treatment was able to reduce the levels of serum LDH and AST in myocardial ischemia/reperfusion rats.

### The effects of Ilexsaponin A on caspase 3, Bcl-2, Bax, p-Akt and Akt expressions in myocardial ischemia/reperfusion rats

**Fig 3:**
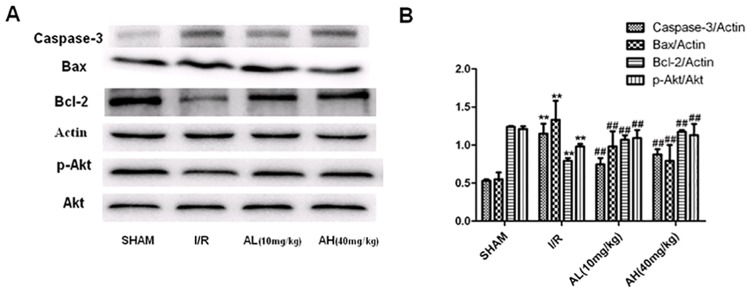
The effect of Ilexsaponin A on caspase-3, cleaved caspase-3, Bax, Bcl-2, p-Akt and Akt expression in injured hearts induced by ischemia/reperfusion. A. Representative blots. B. Relative density refers to the ratio of caspase-3, Bax, Bcl-2 and p-Akt. AL: ilexasaponin A (10mg/kg), AH: ilexasaponin A (40mg/kg), P<0.05 versus CON (**) and versus H/R (##). Bars represent SD; n = 3.

The expressions levels of caspase-3 and bax were significantly increased while the expression levels of bcl-2 were significantly decreased after hypoxia/reoxygenation treatment (Fig 3). Ilexsaponin A treatment was able to decrease the expression levels of caspase-3, bax and increase the expression levels of bcl-2. The expression levels of p-Akt in myocardial ischemia/reperfusion rats were significantly decreased. However, the expression levels of p-Akt in EL and EH group rats were significantly increased after Ilexsaponin A treatment.

### The effects of Ilexsaponin A on cell viability and cellular apoptosis in hypoxia/reoxygenation cardiomyocytes

**Fig 4:**
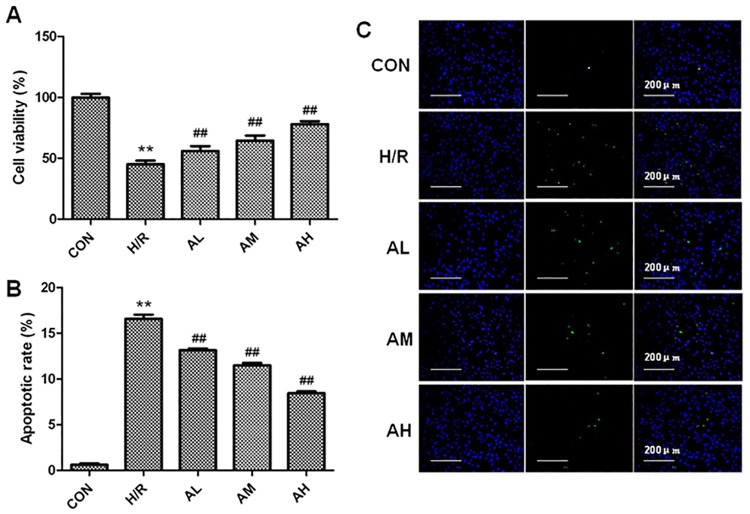
The effect of Ilexsaponin A on cell viability and apoptosis in hypoxia/ reoxygenation treated cardiomyocytes. A. Cell viability was determined by MTT and was calculated by dividing the optical density of samples with the optical density of control. B. Representative photomicrographs of apoptotic cardiomyocytes determined by TUNEL staining. Green fluorescence shows TUNEL-positive nuclei; blue fluorescence shows nuclei of total cardiomyocytes. Bars are 200 μm. C. Percentage of TUNEL-positive nuclei. H/R, hypoxia-reoxygenation treatment group (4h/4h); AL, Ilexsaponin A 10μg/ml treated for 24h; AM, Ilexsaponin A 50μg/ml treated for 24h; AH, Ilexsaponin A 250μg/ml treated for 24h; P<0.05 versus CON (**) and versus H/R (##). Bars represent SD; n = 8.

Compared with the cells in control group, cell viability rate was significantly reduced to 45.10%±3.10% after hypoxia/reoxygenation treatment. Ilexsaponin A pretreatment was able to increase the cell viability rate and this protective effect has a dose-dependent manner. The cell viability rate in EL, EM, EH group cells were respectively 56.09%±3.95%, 64.60%±4.16% and 78.03%±2.56%. Consistently, the cellular apoptosis index was significantly higher after hypoxia/reoxygenation treatment. Ilexsaponin A treatment was able to protect against hypoxia/reoxygenation induced apoptosis (Fig 4).

### The effects of Ilexsaponin A on caspase 3, cleaved caspase-3, bcl-2, bax, p-Akt and Akt expressions in hypoxia/reoxygenation cardiomyocytes

**Fig 5:**
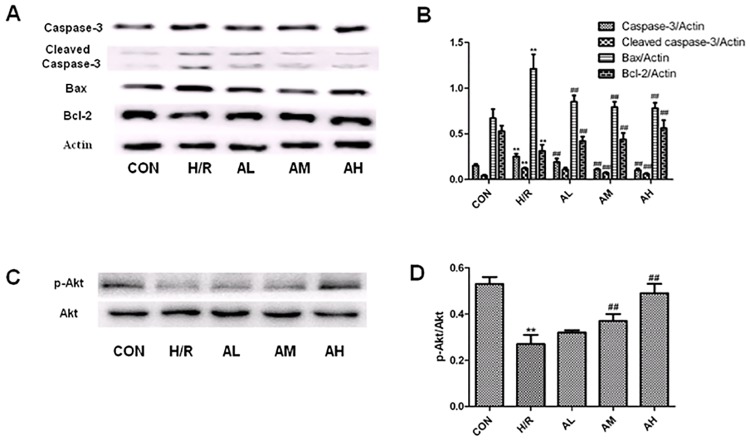
The effect of Ilexsaponin A on caspase-3, cleaved caspase-3, Bax, Bcl-2, p-Akt and Akt expression in hypoxia/ reoxygenation treated cardiomyocytes. A. Representative blots of caspase-3, cleaved caspase-3, Bax and Bcl-2. B. Relative density refers to the ratio of caspase-3, cleaved caspase-3, Bax, Bcl-2 to actin. C. Representative blots of p-Akt and Akt. D. Relative density refers to the ratio of p-Akt to Akt. H/R, hypoxia-reoxygenation treatment group (4h/4h); AL, Ilexsaponin A 10μg/ml treated for 24h; AM, Ilexsaponin A 50μg/ml treated for 24h; AH, Ilexsaponin A 250μg/ml treated for 24h; P<0.05 versus CON (**) and versus H/R (##). Bars represent SD; n = 6.

To examine the anti-apoptotic effects of Ilexsaponin A on hypoxia/reoxygenation cardiomyocytes, we determined several apoptosis associated proteins including caspase-3, cleaved caspase-3, bcl-2 and bax. The expressions levels of caspase-3, cleaved caspase-3 and bax were significantly increased while the expression levels of bcl-2 were significantly decreased after hypoxia/reoxygenation treatment. Ilexsaponin A pretreatment was able to decrease the expression levels of caspase-3, cleaved caspase-3, bax and increase the expression levels of bcl-2 (Fig 5A). Consistent with the results found in in-vivo study, the expression levels of p-Akt in cardiomyocytes after hypoxia-reoxygenation treatment were significantly decreased. However, Ilexsaponin A pretreatment was able to increase the expression levels of p-Akt in a dose-dependent manner (Fig 5B).

## Discussion

Previous studies have shown that the main active components of MDQ are triterpene saponins [6–8], lignan glycosides [18], phenylethanols [19], and other minor compounds [20–21]. Several triterpene saponins have been isolated from MDQ and have been shown to have anti-inflammatory and anti-tumor effects [6,8]. However little is known about their effects on cardiovascular system. We isolated Ilexsaponin A which is one of representative saponins in MDQ (Fig 1). Our study showed that both low dose (10mg/Kg) and high dose (40mg/Kg) of Ilexsaponin A pretreatment were able to decrease the myocardial infarct size in myocardial ischemia/reperfusion rats. In order to confirm this protective effect, we furthermore investigated the levels of markers of myocardial tissue damage, such LDH, AST and CK-MB. The levels of these markers in rat’s serum were significantly increased after myocardial ischemia/reperfusion injury. Ilexsaponin A pretreatment in myocardial ischemia/reperfusion rats was able to decrease the levels of LDH, AST and CK-MB in the serum. These results indicates that Ilexsaponin A protect against myocardial tissue damage in ischemia/reperfusion rats.

It is well documented that myocardial apoptosis contributes to myocardial ischemia/reperfusion injury (MI/RI). Reducing apoptosis could minimize MI/R-induced cardiac damage and therefore slow down or even prevent the occurrence of heart failure [22–24]. Since Ilexsaponin A pretreatment in myocardial ischemia/reperfusion rats could reduce myocardial tissue damage, we hypothesize that Ilexsaponin A might have anti-apoptotic effect on ischemia/reperfusion myocardial cells. We demonstrated that the cell viability was significantly reduced in hypoxia/reoxygenation cardiomyocytes model. Ilexsaponin A pretreatment have beneficial effects on the cell viability after hypoxia/reoxygenation in vitro in a dose-dependent manner. Furthermore, the TUNEL assay showed that Ilexsaponin A pretreatment has been shown to decrease the apoptotic index in hypoxia/reoxygenation cardiomyocytes.

Apoptosis has two distinct cellular pathways: the extrinsic pathway (or death receptor pathway) and the intrinsic pathway (also called mitochondrial pathway). Following activation of either the intrinsic or extrinsic pathways of the apoptotic cascade, initiator caspases cleave and activate the executioner caspases-3, -6 or -7 [25]. Pro-caspase-3 becomes an active enzyme when two cleaved monomers come together to form an active dimmer. Our study demonstrated that the expression levels of caspase-3 and cleaved caspase-3 were significantly increased in hypoxia/reoxygenation cardiomyocytes and Ilexsaponin A pretreatment was able to inhibit the activation of caspase-3. It is well known that bcl-2 protein family plays a important role in regulating apoptosis process. Bcl-2 members are localized in the mitochondria and have either proapoptotic (Bax, Bak, Bid, and Bim) or anti-apoptotic (Bcl-2, Bcl-xL, and Bcl-W) functions [26–28]. Ilexsaponin A pretreatment increase the expression levels of the anti-apoptotic protein Bcl-2 and increase the expression levels of pro-apoptotic effector protein Bax. These results confirmed the Ilexsaponin A could attenuate apoptosis on ischemia/reperfusion myocardial cells. Phosphatidylinositol-3 kinase/protein kinase B (PI3K/Akt), an intracellular signaling pathway, is involved in cell survival, apoptosis, growth, energy metabolism and migration [29]. Numerous studies have shown that Akt activation exerts beneficial effects on ischemic hearts [30–32]. Cosistently, the in-vivo and the in-vitro studies demonstrated that the myocardial beneficial effects of Ilexsaponin A was associated with the activation of Akt pathway.

In conclusion, our study demonstrated that Ilexsaponin A could protect against myocardial damage in myocardial ischemia/reperfusion rats and inhibit hypoxia/reoxygenation induced cardiomyocytes apoptosis via Akt activation. These results imply that Ilexsaponin A has potential as a novel cardioprotective agent for treating MI/RI.

## Supporting information

S1 Fig HR-ESI-MS and IR spectra of Ilexsaponin A. A. HR-ESI-MS spectra of Ilexsaponin A. B. IR spectra of Ilexsaponin A.
(TIF)

S2 Fig ^1^H-NMR and ^13^C-NMR spectra of Ilexsaponin A. A. ^1^H-NMR spectra of of Ilexsaponin A. B. ^13^C-NMR spectra of Ilexsaponin A.
(TIF)
