# Supplementary figures and images for: Ilexsaponin A attenuates ischemia-reperfusion-induced myocardial injury through anti-apoptotic pathway
Source: PLoS One. 2017 Feb 9;12(2):e0170984. doi: 10.1371/journal.pone.0170984 (PMC5300190; doi:10.1371/journal.pone.0170984)

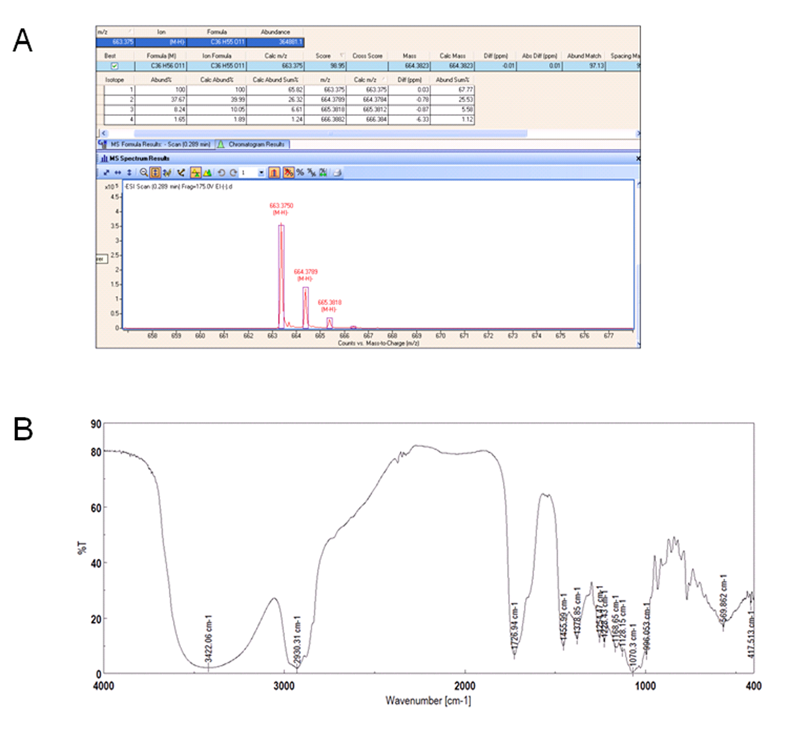

Supplement: S1 Fig — A. HR-ESI-MS spectra of Ilexsaponin A. B. IR spectra of Ilexsaponin A. (TIF) [file pone.0170984.s001.tif]

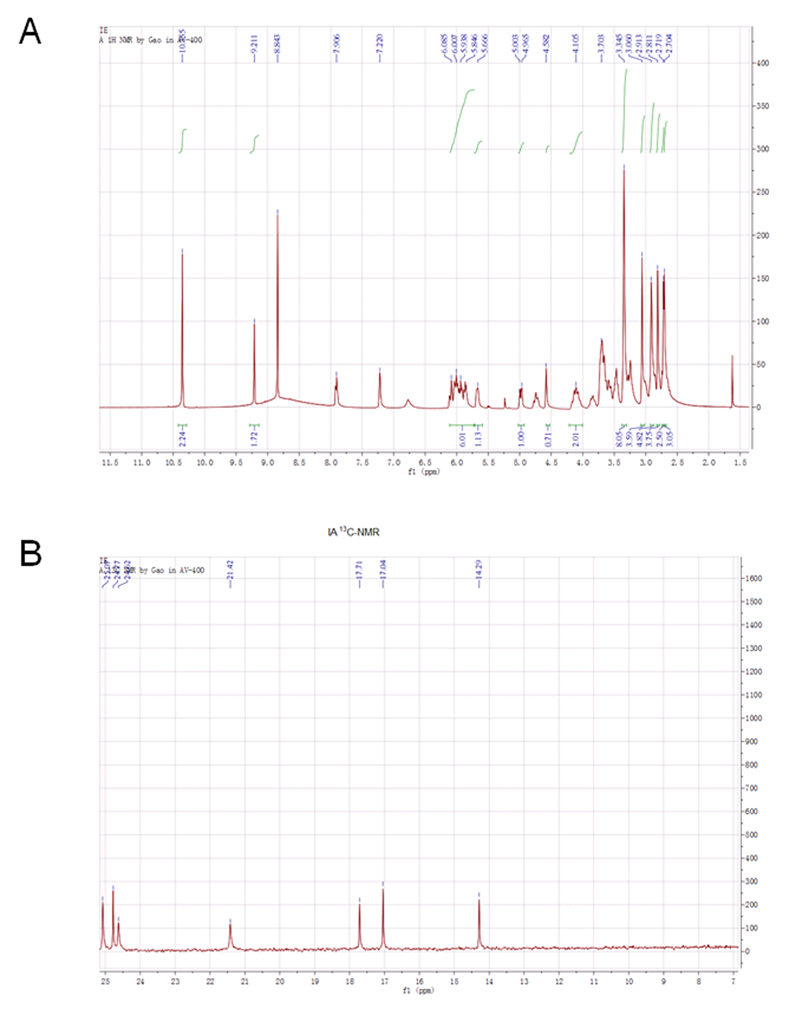

Supplement: S2 Fig — A. 1H-NMR spectra of of Ilexsaponin A. B. 13C-NMR spectra of Ilexsaponin A. (TIF) [file pone.0170984.s002.tif]
